# *Atractylodes macrocephala* Koidz. volatile oil relieves acute ulcerative colitis *via* regulating gut microbiota and gut microbiota metabolism

**DOI:** 10.3389/fimmu.2023.1127785

**Published:** 2023-05-02

**Authors:** Hao Cheng, Dandan Zhang, Jing Wu, Juan Liu, Yuzhu Tan, Wuwen Feng, Cheng Peng

**Affiliations:** ^1^ State Key Laboratory of Southwestern Chinese Medicine Resources, School of Pharmacy, Chengdu University of Traditional Chinese Medicine, Chengdu, China; ^2^ The Ministry of Education Key Laboratory of Standardization of Chinese Herbal Medicine, School of Pharmacy, Chengdu University of Traditional Chinese Medicine, Chengdu, China; ^3^ Hospital of Chengdu University of Traditional Chinese Medicine, Chengdu, China

**Keywords:** ulcerative colitis, *Atractylodes macrocephala* Koidz. volatile oil, gut microbiota, bile acids, tryptophan metabolism, retinoic acid metabolism

## Abstract

**Background:**

*Atractylodes macrocephala* Koidz. (AM) is a functional food with strong ant-colitis activity. AM volatile oil (AVO) is the main active ingredient of AM. However, no study has investigated the improvement effect of AVO on ulcerative colitis (UC) and the bioactivity mechanism also remains unknown. Here, we investigated whether AVO has ameliorative activity on acute colitis mice and its mechanism from the perspective of gut microbiota.

**Methods:**

Acute UC was induced in C57BL/6 mice by dextran sulfate sodium and treated with the AVO. Body weight, colon length, colon tissue pathology, and so on were assessed. The gut microbiota composition was profiled using 16s rRNA sequencing and global metabolomic profiling of the feces was performed. The results showed that AVO can alleviate bloody diarrhea, colon damage, and colon inflammation in colitis mice. In addition, AVO decreased potentially harmful bacteria (*Turicibacter*, *Parasutterella*, and *Erysipelatoclostridium*) and enriched potentially beneficial bacteria (*Enterorhabdus*, *Parvibacter*, and *Akkermansia*). Metabolomics disclosed that AVO altered gut microbiota metabolism by regulating 56 gut microbiota metabolites involved in 102 KEGG pathways. Among these KEGG pathways, many metabolism pathways play an important role in maintaining intestine homeostasis, such as amino acid metabolism (especially tryptophan metabolism), bile acids metabolism, and retinol metabolism.

**Conclusion:**

In conclusion, our study indicated that AVO can be expected as novel prebiotics to treat ulcerative colitis, and modulating the composition and metabolism of gut microbiota may be its pharmacological mechanism.

## 1 Introduction

As an inflammatory bowel disease, ulcerative colitis (UC) is characterized by tissue inflammation occurring in the rectum and steadily spreading to the colon (1, 2). UC has no gender difference, and the peak age of onset of it is 30 to 40 years old (1). The incidence and prevalence of UC in the populations of Western Europe and North America are higher than that in Asia and the incidence of UC is also increasing in Asia in recent years (3). UC brings a heavy economic burden on UC patients and patients with UC have a higher probability to develop intestinal cancer (4). Although there are many drugs clinically used to treat UC, such as 5-aminosalicylic acid, tumor necrosis factor-*α* antibodies, and immunomodulators, these drugs often have diverse side effects, such as infection and fever (5). Some severe UC patients even require surgery, but various complications may occur after surgery, such as refractory toxic megacolon, perforation, and persistent severe large bowel bleeding (5, 6). Accordingly, it is urgent to find a safe, effective, and economical therapeutic measure for UC.

*Atractylodes macrocephala* Koidz. (AM) is one of the best-known herbal medicinal in China, and AM is a component of more than 117 healthy foods (7). AM has various biological activities, including but not limited to anti-inflammatory activity, gastrointestinal protective activity, hypoglycemic activity, and anticancer activity (7). A larger number of modern researches have emphasized the beneficial effect of AM on UC (7, 8). However, the research on the specific material basis for AM maintaining intestinal homeostasis is still defective. The main chemical constituents of AM are terpenoids and their glycosides, alkynes and their glycosides, flavonoids and their glycosides, steroids, and polysaccharides (7, 9). AM volatile oil (AVO, main terpenoids) is the main ingredient of AM with well anti-inflammatory activities (10). Accordingly, we speculated that AVO may be a component of AM with therapeutic effects on UC.

The etiology of UC is complex. Among these etiologies, the dysregulated gut microbiota (GM) has been considered a key contributing factor and GM has been considered an important therapeutic target for UC due to the inextricable links between GM and intestine barriers (11). GM inhabits the intestine in a symbiotic relationship and influences intestine homeostasis by various mechanisms. In one direction, some symbiotic microorganisms colonizing the mucus layer maintain intestine homeostasis by inhibiting pathogen colonization (12). In the other direction, the intestinal barrier cells can respond to commensal bacteria-derived signals and then induce antimicrobial and immunoregulatory responses (13). In addition, GM metabolites are closely interrelated to intestine homeostasis as well, for example, tryptophan microbial derivatives can maintain intestine barrier integrity and improve intestinal inflammation by triggering aryl hydrocarbon receptor (AHR) expressed in intestinal epithelial cells (14). In recent years, many researchers have shown that the bioactivity of AVO may be largely attributed to the regulation of AVO on GM (15). Considering the above facts, we supposed that if AVO has anti-colitis activity, the underlying mechanism may be closely correlated to regulating GM and GM metabolism.

In our study, we first investigated whether AVO has curative efficacy for UC and the mechanism from the perspective of GM. An acute UC mouse model induced by dextran sulfate sodium (DSS) was chosen as the experimental subject. Phenotypic indicators of UC mice were used to evaluate the curative efficacy of AVO, such as bloody diarrhea score, colon histopathology, and colonic immune-related cytokines. The GM composition and metabolism of UC mice were determined by bacterial 16S rRNA sequencing and ultra-performance liquid chromatography-mass spectrometry (UPLC-MS), respectively. Our study will be of significance for a more comprehensive understanding of the anti-colitis mechanism of AVO and developing AVO as novel prebiotics to treat UC.

## 2 Materials and methods

### 2.1 Reagents and materials

AM was purchased from the flagship store of Wenwu Mountain (Pan’an County, Jinhua City, Zhejiang Province, China). DSS (36-50 kDa) was bought from Yeasen Biotech Co., Ltd. (Shanghai, China). MS-grade formic acid was purchased from CNW (Shanghai, China), methanol was purchased from Fisher Chemical (USA), acetonitrile was purchased from Fisher Chemical (USA), and 2-propanol was purchased from Merck (Germany).

### 2.2 The extraction of *Atractylodes macrocephala* volatile oil

AVO was extracted according to the way described elsewhere (15). Briefly, 350 g AM was divided into 7 equal parts and then transferred into seven 1000 ml flasks with 500 ml water, respectively. 2.0 ml AVO was eventually gathered after heating and refluxing extraction for 8 h. To prevent the degradation of compounds, the freshly extracted AVO was store in 4 °C condition.

### 2.3 Ingredient identification of *Atractylodes macrocephala* volatile oil

**Figure 1:**
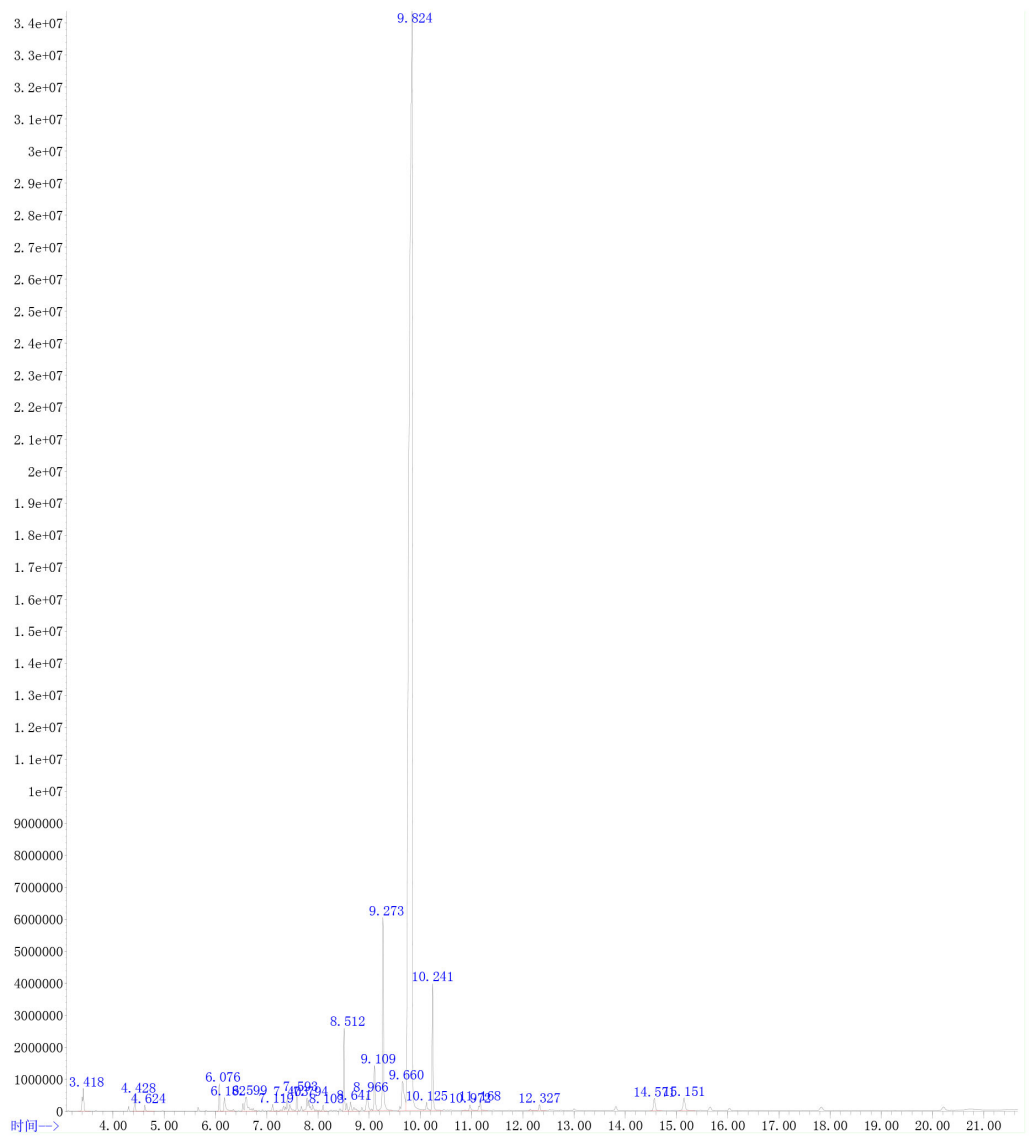
The GC-MS characteristics map of *Atractylodes macrocephala* Koidz.volatile oil (AVO).

The extracted AVO was characterized according to the way that has been described elsewhere (15). Briefly, gas chromatography (GC)-MS analysis technology was used to identify the compounds of AVO after mixing 20 μl AVO and 980 μl n-hexane and drying with anhydrous sodium sulfate. The 7890A-5975C system (Agilent, USA) was adopted to carry out GC-MS analysis. The column was Agilent 19091S-433 HP-5MS (Agilent, USA). The carrier gas was helium and the volume flow of helium was set at 1.0 ml/min. The initial column temperature was 80 °C, subsequently increased to 230°C at 15°C/min and remained for 2 min, then increased to 250°C at 3°C/min, and finally increased to 270°C at 10°C/min. The injection volume was 1 μl and the flow rate was 1.0 ml/min. The ion source temperature was 230°C, and the ion scan range of MS was full scan. The constituents of AVO were identified by the comparison with known constituents stored in the National Institute of Standards and Technology Library 14 (NIST14). The percentage of each component was calculated by the method of peak area normalization based on the total ion flow chart. The result of GC-MS was shown in **Figure 1** and **Table 1**.

**Table 1:** The characteristics ingredients of AVO.

Component name	Molecular formula	Peak time (min)	Proportion (%)
trans-*β*-Ocimene	C_10_H_16_	3.416	0.73
5-Pentylcyclohexa-1, 3-diene	C_11_H_18_	4.622	0.15
2-Methoxy-4-vinylphenol	C_9_H_10_O_2_	6.183	0.52
2, 4, 6-Cycloheptatrien-1-one	C_7_H_6_O	6.600	1.02
(+)-*β*-Funebrene	C_15_H_24_	7.119	0.16
4, 8-Methanoazulene	C_15_H_24_	7.402	0.82
N-(2-Furanylmethyl)-1, 2-dimethyl-N-[3-(1-methylethoxy) propyl]-1*H*-imidazole-5-methanamine	C_17_H_27_N_3_O_2_	7.592	0.41
(+)-Ledene	C_15_H_24_	7.794	0.98
(+)-*β*-Himachalene	C_15_H_24_	8.102	0.15
Espatulenol	C_15_H_24_O	8.511	1.80
(+)-Ledene	C_15_H_24_	8.642	0.44
Spathulenol	C_15_H_24_O	8.967	0.72
3-Butyl-1(3*H*)-isobenzofuranone	C_12_H_14_O_2_	9.110	1.17
3-Butylidene-1(3*H*)-isobenzofuranone	C_12_H_12_O_2_	9.275	4.74
(*Z*)-Butylidenephthalide	C_12_H_12_O2	9.658	1.69
Atractylone	C_15_H_20_O	9.823	78.82
(*Z*)-3-Butylidene-4, 5-dihydroisob	C_12_H_14_O_2_	10.240	2.72
5, 7-Dimethoxy-2-methyl-2, 3-dihydro-1*H*-inden-1-one	C_12_H_14_O_3_	10.970	0.20
Oleamide	C_18_H_35_NO	15.150	0.61

### 2.4 Animals and treatments

**Figure 2:**
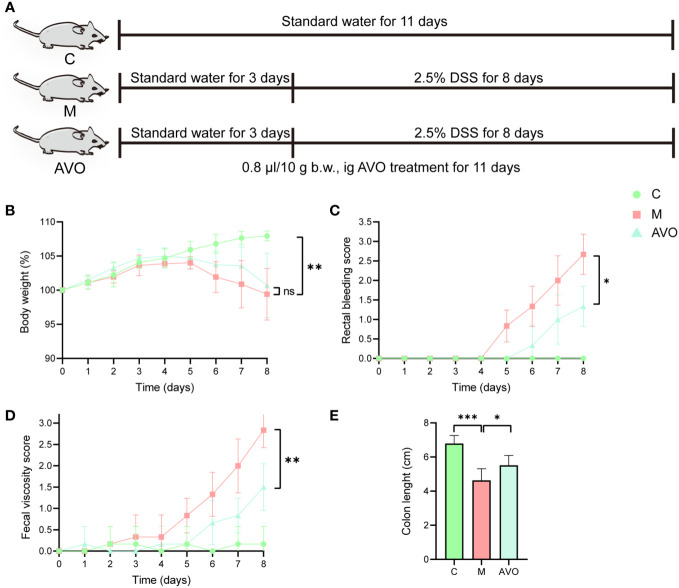
The curative effect of AVO on UC mice. **(A)** The method of animal grouping and treatment. **(B)** The change of body weight of each group after DSS challenge and AVO treatment. **(C)** The rectal bleeding score of each group after DSS challenge and AVO treatment. **(D)** The fecal viscosity score of each group after DSS challenge and AVO treatment. **(E)** The colon length of each group after DSS challenge and AVO treatment. *P ≤ 0.05, **P ≤ 0.01, ***P ≤ 0.001, and ns means P > 0.05.

The animal experiment was authorized by the Committee on the Ethics of Animal Experiments of Chengdu University of Traditional Chinese Medicine (No. 2021-73). 20.0 ± 2.0 g C57BL/6J mice (male, n = 18) were supplied by the Beijing Speifu Biotechnology Co., Ltd. (China, SCXK (jing) 2019-0010). Mice were housed in a standard SPF environment with temperature (22 ± 2°C) and humidity (50 ± 10%), and mice can freely receive a standard diet and water. After acclimatization for 5 days, mice were divided randomly into the following three groups (each group n = 6): the control (C) group, the model (M) group, and the AVO group. The C group mice freely receive a standard diet and water for 11 days. The M group mice received the stimulation of 2.5% DSS (2.5 g DSS dissolved in 100 ml drinking water) from the 4^th^ day to the 11^th^ day. The AVO group mice received the stimulation of 2.5% DSS from the 4^th^ day to the 11^th^ day and AVO treatment (0.8 μl/10 g b.w., ig) for 11 days. The AVO was dissolved in 0.1% sodium carboxymethyl cellulose solution (15). **Figure 2A** showed the method of animal grouping and treatment.

### 2.5 The records of body weights, diarrhea score, rectal bleeding score, and collection of samples

With the experiment going on, at a fixed time per day, the body weight was recorded, and diarrhea and bloody stool were evaluated based on the rules that have been described in our previous study (8). To prevent bleeding from affecting results and the inability to retrieve stool due to diarrhea, we gathered the feces on the 7^th^ and 8^th^ days, and the feces was used to carry out the 16S-rRNA sequencing and global metabolomics analysis, respectively. The collected fecal samples were stored at -80°C. At the end of the experiment, we measured the mice’s colon length and cut the colon into two segments. The two segments colon were used for histopathological and immune-related cytokines examination. The colon segment for histopathological examination was fixed by 4% paraformaldehyde, and the other colon segment for immune-related cytokines examination was stored at -80°C.

### 2.6 Histopathological and immune-related cytokines measurement

After paraffin embedding and slicing, the colon segment fixed by 4% paraformaldehyde received hematoxylin and eosin (H&E) staining. The other segment colon was weighed, mixed with RIPA lysis buffer (w/v, 9 μl/mg), ground with a tissue grinder, and centrifuged at 4°C for 5 min. Subsequently, the supernatant was gathered for the measurement of immune-related cytokines in colon tissue. The contents of IL-2, IL-4, IL-17, and INF-*γ* in the supernatant were detected by the BD Cytometric Bead Array Mouse Th1/Th2/Th17 Cytokine Kit (BD Biosciences, USA). Finally, the concentrations of cytokines were calculated by FCAP v3.0 software.

### 2.7 Genomic DNA extraction and 16S-rRNA sequencing of fecal samples

The microbial DNA of fecal samples was extracted by the E.Z.N.A.^®^ soil DNA Kit (Omega Bio-tek, USA) and amplified by ABI GeneAmp^®^ 9700 PCR thermocycler (ABI, USA). The primer pairs used for amplification were 338F and 806R. Extracted and purified the amplified product by using the AxyPrep DNA Gel Extraction Kit (Axygen Biosciences, Union City, CA, USA). The purified PCR was quantified via using QuantusTM Fluorometer (Promega, USA) and was performed on an Illumina MiSeq PE300 platform/NovaSeq PE250 platform (Illumina, San Diego, USA). The raw 16S rRNA gene sequencing reads were demultiplexed, quality-filtered by FASTP (version 0.20.0), and merged by FLASH (version 1.2.7). The conditions for quality filtering and merging have been described elsewhere (16). Briefly, at any site with an average quality score of < 20 over a 50 bp sliding window, the 300 bp reads were truncated, and reads containing ambiguous characters were discarded. Overlapping sequences longer than 10 bp were assembled and the mismatch rate of the overlapping sequences was less than 0.2. Samples were distinguished according to the barcode and primers. Operational taxonomic units (OTUs) with a 97% similarity cutoff were clustered by UPARSE software (version 7.1) and analyzed by using a confidence threshold of 0.7 in RDP Classifier (version 2.2).

### 2.8 Global metabolomics analysis of mice fecal sample

50.0 mg fecal sample was weighed and mixed with 400.0 µl of extract (methanol: acetonitrile = 1:1 (v: v)) in a 2.0 ml Ep tube. Freeze-grinded at -10°C for 6 min with a cryo-tissue grinder and then ultrasound extracted at 5°C for 30 min. Subsequently, centrifuged at 4 °C, 13000 g for 15 min, and gathered the supernatant for onboard analysis. UPLC-MS analysis was carried out by the UHPLC-Q Exactive HF-X system (Thermo Scientific, USA). The used column in the UHPLC was the ACQUITY UPLC HSS T3 (Waters, USA). The mobile phase elution methods are shown in **Table S1**. In positive and negative ion modes, the spray voltages of MS were 3500 V and -3500 V, respectively. The MS scanning range was 70-1050 m/z. Collected the MS signals and identified characteristic peaks by the Progenesis QI (Waters Corporation, USA). Subsequently, the metabolites were identified by matching the MS information with the metabolites database (e.g., HMDB database, scripps database).

The extracted metabolites’ data was subsequently analyzed based on the following ways: (1) eliminated metabolites with more than 20% missing values within a group; (2) missing values were simulated with minimal values; (3) the data normalization method was sum; (4) eliminated variables whose RSD ≤ 30%; (5) Use log10 as the value method. Principal component analysis (PCA) analysis showed dispersion among all samples. Variables with VIP > 1.0 and *P* < 0.05 in the orthogonal partial least square’s discriminant analysis (OPLS-DA) model were shown by volcano plots. Furthermore, only these GM metabolites meeting the requirements of statistical analysis and dose-effect relationship among all groups were screened out by analyzing all identified metabolites. The enrichment analysis of all identified metabolites using the KEGG database (database version: keg_v2021.09.18) as the enrichment background was performed. The enrichment analysis of the screened metabolites was performed in Metaboanalyst 5.0 online platform.

### 2.9 Statistical analysis

The data were exhibited as mean ± standard variance (Ace and Chao index were presented as min to max). Statistical significance of the data was determined based on the following ways by using GraphPad Prism 9.0.0 software (La Jolla, USA): (1) If normally distributed and variance is homogeneous, the statistical significance of data was assessed by ANOVA followed by Dunnett’s multiple comparisons test; (2) If normally distributed but the variance isn’t homogeneous, the statistical significance of data was assessed by Brown-Forsythe and Welch’s ANOVA followed by Dunnett’s multiple comparisons test; (3) If didn’t normally distributed, the statistical significance of data was assessed by Kruskal-Wallis test followed by Dunn’s multiple comparisons test; (4) The Mann-Whitney test was performed for immune-related cytokine, diarrhea score, and rectal bleeding score statistical analysis. *P* < 0.05 was considered to be statistically significant. *P < 0.05, **P < 0.01, ***P < 0.001, and ns means *P* > 0.05.

## 3 Results

### 3.1 Ingredients of *Atractylodes macrocephala* volatile oil

As shown in **Table 1** and **Figure 1**, 19 ingredients were successfully identified in the AVO by GC-MS analysis. The main components are atractylone (78.82%), 3-butylidene-1(3*H*)-isobenzofuranone (4.74%), (*Z*)-3-butylidene-4, 5-dihydroisob (2.72%), espatulenol (1.80%), (*Z*)-butylidenephthalide (1.69%), and 3-butyl-1(3*H*)-isobenzofuranone (1.17%). The 6 compounds represent 90.94% of AVO and atractylone was the representative ingredient of AVO.

### 3.2 The effect of *Atractylodes macrocephala* volatile oil on colitis mice

**Figure 3:**
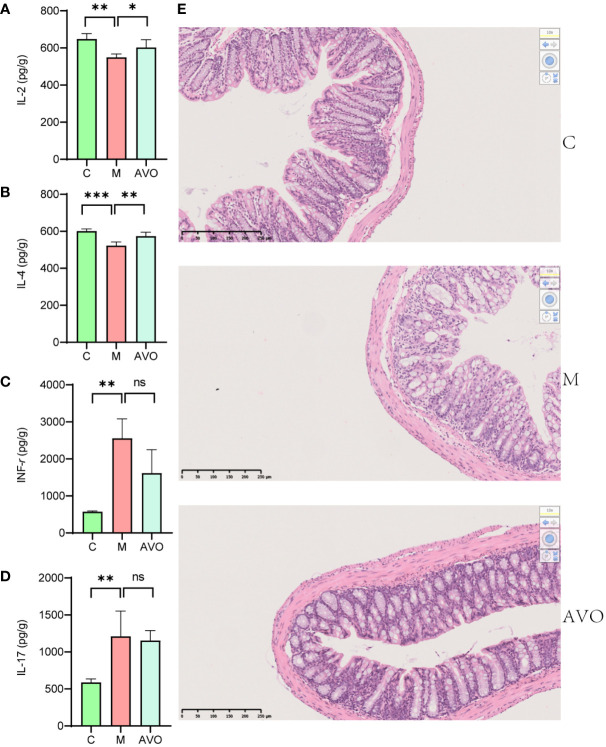
The curative effect of AVO on UC mice. **(A–D)** The levels of IL-2, IL-4, INF-*γ*, and IL-17 in colon tissue. **(E)** HE staining results in UC mice colon tissue. *P ≤ 0.05, **P ≤ 0.01, ***P ≤ 0.001, and ns means P > 0.05.

To evaluate the curative effect of AVO on UC, we recorded the phenotype of UC mice during the DSS challenge and AVO treatment. In our study, the DSS challenge resulted in significant body weight loss, and AVO treatment prevented DSS-induced weight loss although there was no statistical significance (**Figure 2B**). In addition, compared with the C group, we observed significant bloody diarrhea in the M group mice, and AVO treatment significantly relieved bloody diarrhea (**Figures 2C, D**). Through anatomical analysis, we found that the mice’s colon length was shortened after DSS stimulation, and AVO treatment significantly relieved DSS-induced colon shortening (**Figure 2E**). In addition, we detected immune-related cytokines in mice colon tissue. As shown in **Figures 3A–D**, the content of anti-inflammatory cytokines IL-2 and IL-4 was decreased and the content of proinflammatory cytokines IL-17 and INF-*γ* was promoted in the M group mice. However, the content of IL-2 and IL-4 was increased after AVO treatment. HE staining results showed that compared with the C group, DSS-challenge resulted in obvious intestinal tissue damage which was mainly manifested as the destroyed crypt structure, the destroyed colonic mucosa, and the increased inflammatory infiltration. Nevertheless, the alleviated intestinal tissue damage was observed in AVO treatment mice (**Figure 3E**).

### 3.3 The effect of *Atractylodes macrocephala* volatile oil and DSS on gut microbiota composition

**Figure 4:**
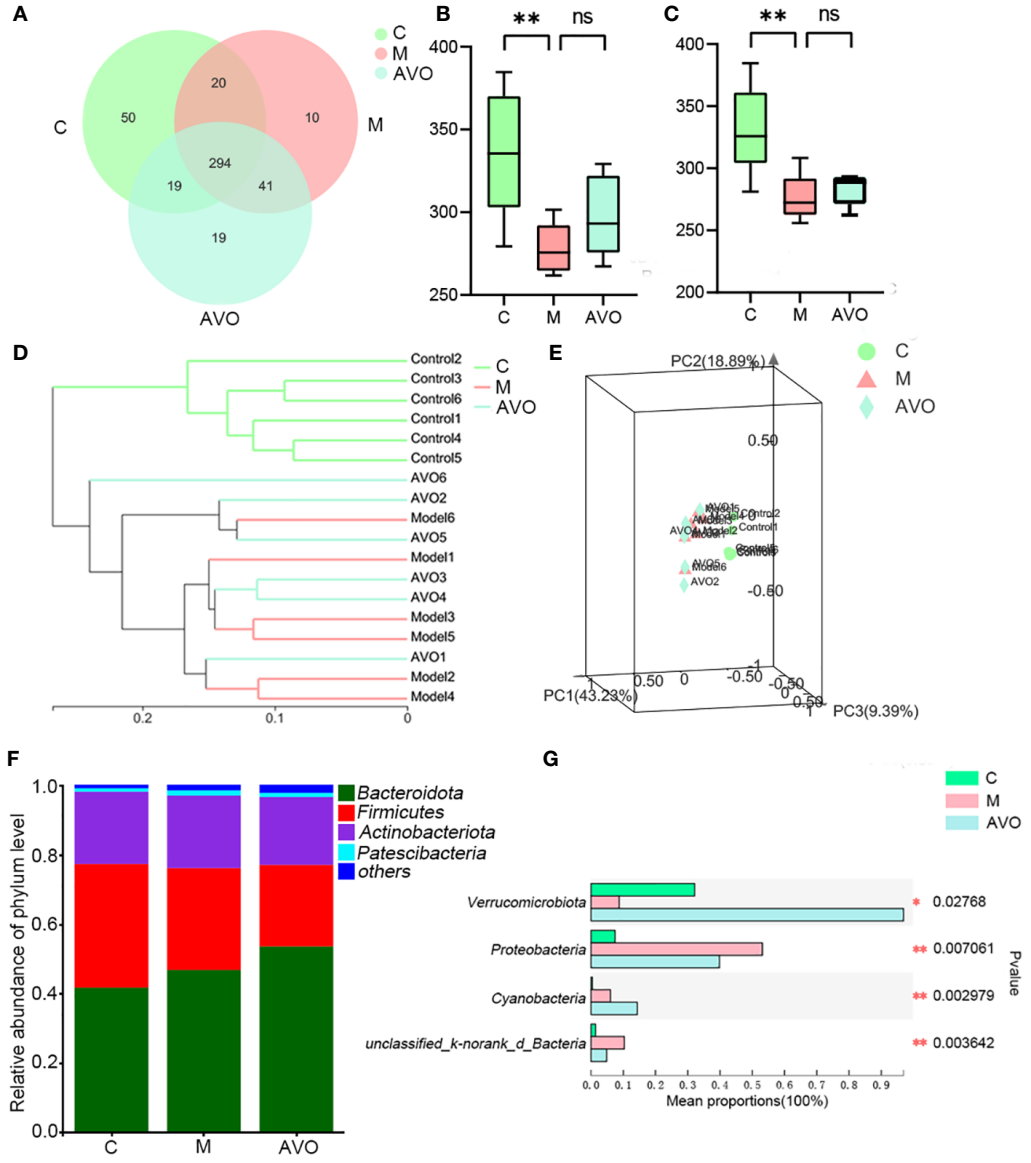
The effect of AVO and DSS on GM overall structure in UC mice. **(A)** The Venn diagram among three groups. **(B)** Ace index. **(C)** Chao index. **(D)** Hierarchical clustering among all fecal samples. **(E)** 3D PCoA shows the similarities and differences among the three groups. **(F)** Histogram among three groups. **(G)** The differential bacteria among three groups at the phylum level. *P ≤ 0.05, **P ≤ 0.01, and ns means P > 0.05.

To determine whether the bioactivity of AVO on UC is closely correlated to regulating GM, the GM composition of mice fecal samples was determined and analyzed. The changes in GM composition after the DSS challenge and AVO treatment are shown in **Figure 4** and **Figure S1**. The Pan and Core curves showed the number of total OTUs and shared OTUs contained in all samples, respectively. With an increase in the number of samples, the flattened curve indicated that the sample size for sequencing is reasonably up to standard (**Figures S1A, B**). After OTU clustering, 453 OTUs were clustered in all fecal samples and 294 OTUs were clustered in all groups. Among these OTUs, 50, 10, and 19 OTUs were clustered alone in the C, M, and AVO groups, respectively (**Figure 4A**). Ace and Chao index suggested that the DSS challenge led to a significant reduction in GM richness (**Figures 4B, C**). However, AVO has no significant effect on the DSS-induced GM richness reduction. In addition, the hierarchical clustering and the 3D principal coordinates analysis (PCoA) plot showed that DSS challenge led to significant change of GM overall composition (**Figures 4D, E**). Since M and AVO groups are not clearly separated, suggesting that the change of specific microorganisms rather than the overall GM structure is associated with the efficacy of AVO. The detected microorganisms in all samples mainly belonged to *Firmicutes*, *Bacteroidota*, *Actinobacteriota*, and *Patescibacteria* (**Figure 4F**). Compared with the C group, the DSS challenge led to decreased *Verrucomicrobia* and increased *Proteobacteria*. *Verrucomicrobia* was increased and *Proteobacteria* was decreased after AVO oral administration compared with the UC mice (**Figure 4G**).

**Figure 5:**
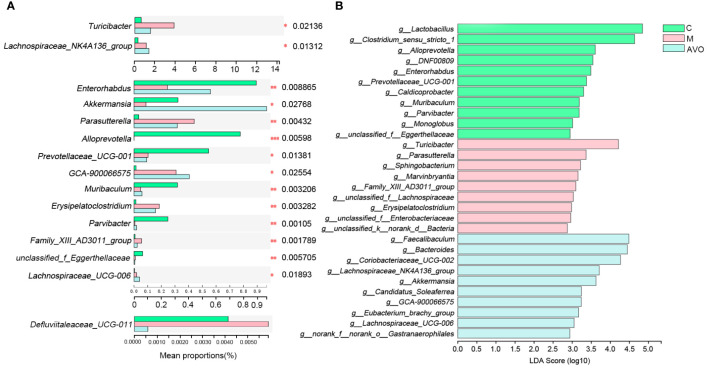
The change of GM after AVO treatment and DSS stimulation at the genus level. **(A)** The differential bacteria. **(B)** The linear discriminant analysis of effect size. *P ≤ 0.05, **P ≤ 0.01, ***P ≤ 0.001, and ns means P > 0.05.

In addition to analyzing the overall structure, we dug deeper into the changes of GM at the genus level. 5 genera *Parasutterella*, *Erysipelatoclostridium*, *Turicibacter*, *Defluviitaleaceae_UCG-011*, and *Family_XIII_AD3011_group* were enriched and 3 genera *Enterorhabdus*, *Akkermansia*, and *Parvibacter* were decreased after DSS intervention. Compared with the M group, *Parasutterella*, *Erysipelatoclostridium*, *Defluviitaleaceae_UCG-011*, *Turicibacter*, and *Family_XIII_AD3011_group* was decreased and *Enterorhabdus*, *Akkermansia*, and *Parvibacter* were increased after AVO treatment (**Figure 5A**). In addition, the linear discriminant analysis of effect size (LEfSe) identified the dominant microorganisms which may supply a great contribution to the intergroup differences (**Figure 5B**), *Turicibacter*, *Family_XIII_AD3011_group*, *Parasutterella*, *Sphingobacterium*, *Marvinbryantia*, *Lachnospiraceaein*, *Erysipelatoclostridium*, and *Enterobacteriaceae* had high LDA score and were the key types of bacteria that may be responsible for the altered GM composition in the M group. In addition, *Faecalibaculum*, *Bacteroides*, *Akkermansia*, *Coriobacteriaceae_UCG-002*, *Lachnospiraceae_NK4A136_group*, *Gastranaerophilales*, *Candidatus_Soleaferrea*, *GCA-900066575*, *Eubacterium_brachy_group*, and *Lachnospiraceae_UCG-006* had high LDA score in the AVO group. The result indicated that these microorganisms greatly contribute to the differences in GM composition between the AVO group and the other groups.

### 3.4 The modification effect of *Atractylodes macrocephala* volatile oil and DSS on gut microbiota metabolism

**Figure 6:**
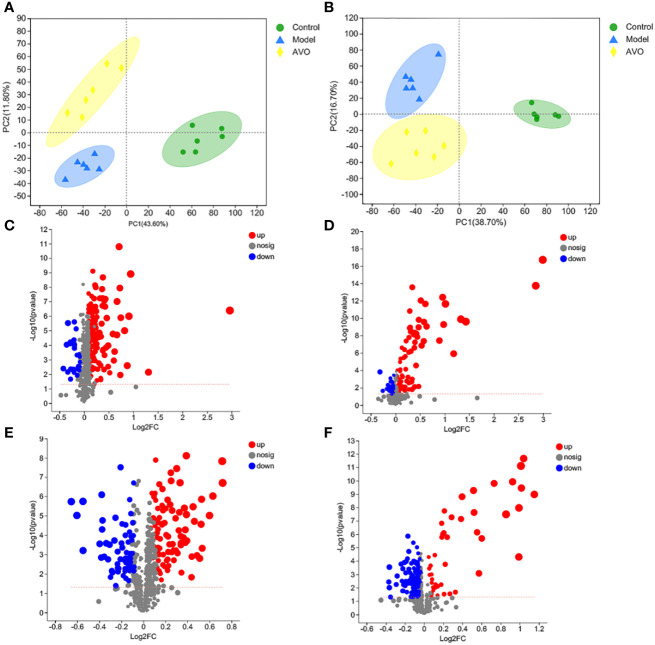
DSS and AVO modulated GM metabolism. **(A)** PCA score plots of all samples in positive ion mode. **(B)** PCA score plots of all samples in negative ion mode. **(C)** The volcano map reflects these variables between the C group and the M group in positive ion mode. **(D)** The volcano map reflects these variables between the AVO group and the M group in positive ion mode. **(E)** The volcano map reflects these variables between the C group and the M group in negative ion mode. **(F)** The volcano map reflects these variables between the AVO group and the M group in negative ion mode.

In addition to GM composition, GM metabolism may be correlated to intestinal homeostasis and the therapeutic effect of AVO on UC, so we performed the global metabolomics analysis of mice fecal samples. As shown in the PCA score plot (**Figures 6A, B**, **Figures S2A–D**), the three groups were separated from each other in both positive and negative ion modes. These results suggested that DSS and AVO significantly affected GM metabolism. These variables meet the standard described in the 2.6 section were shown by volcano plot and these variables are conducive to the separation between the two groups (**Figures 6C–F**).

**Figure 7:**
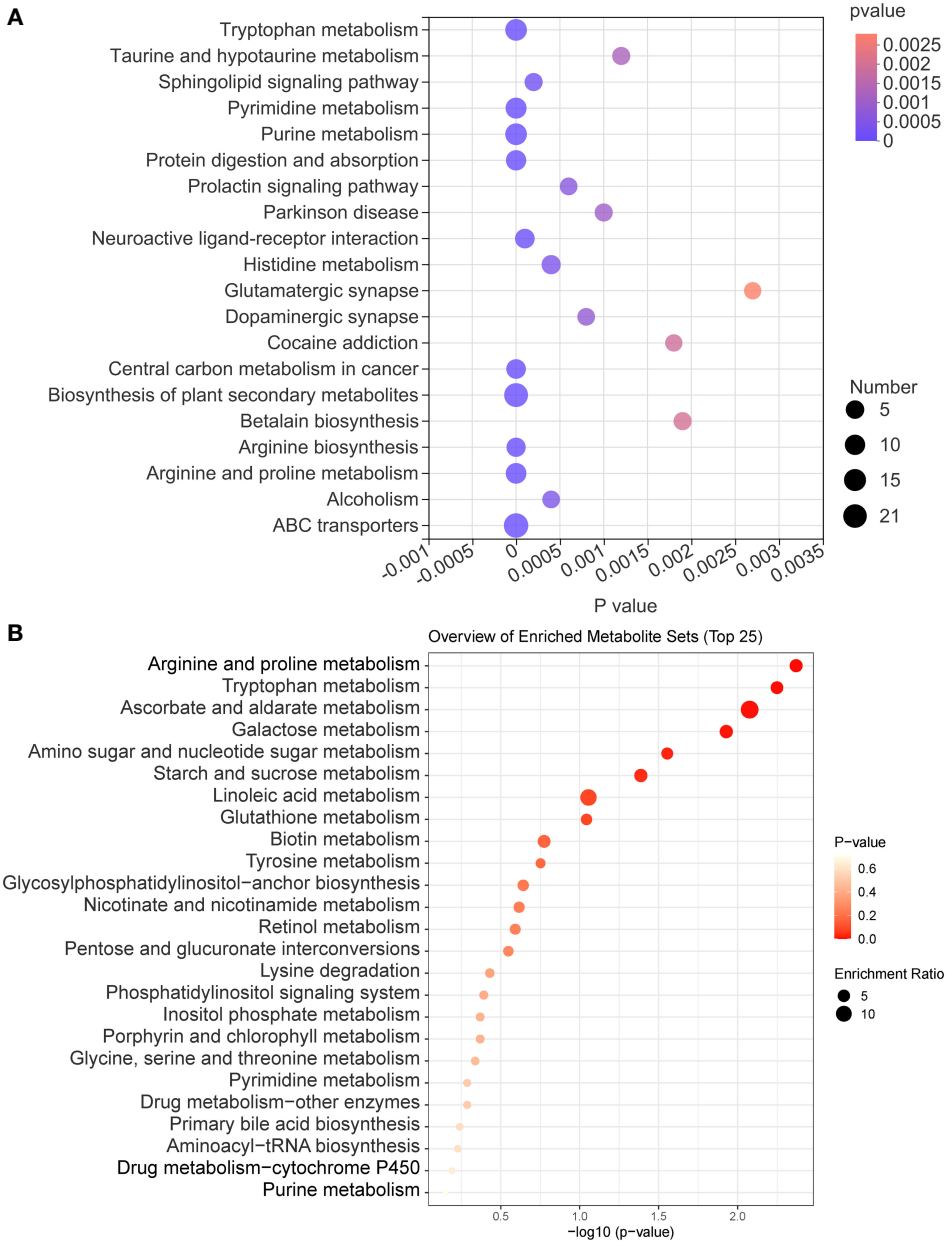
The KEGG enrichment analysis of GM metabolites. **(A)** The KEGG enrichment analysis bubble diagram of all identified metabolites. **(B)** The KEGG enrichment analysis bubble diagram of 56 GM metabolites.

**Figure 8:**
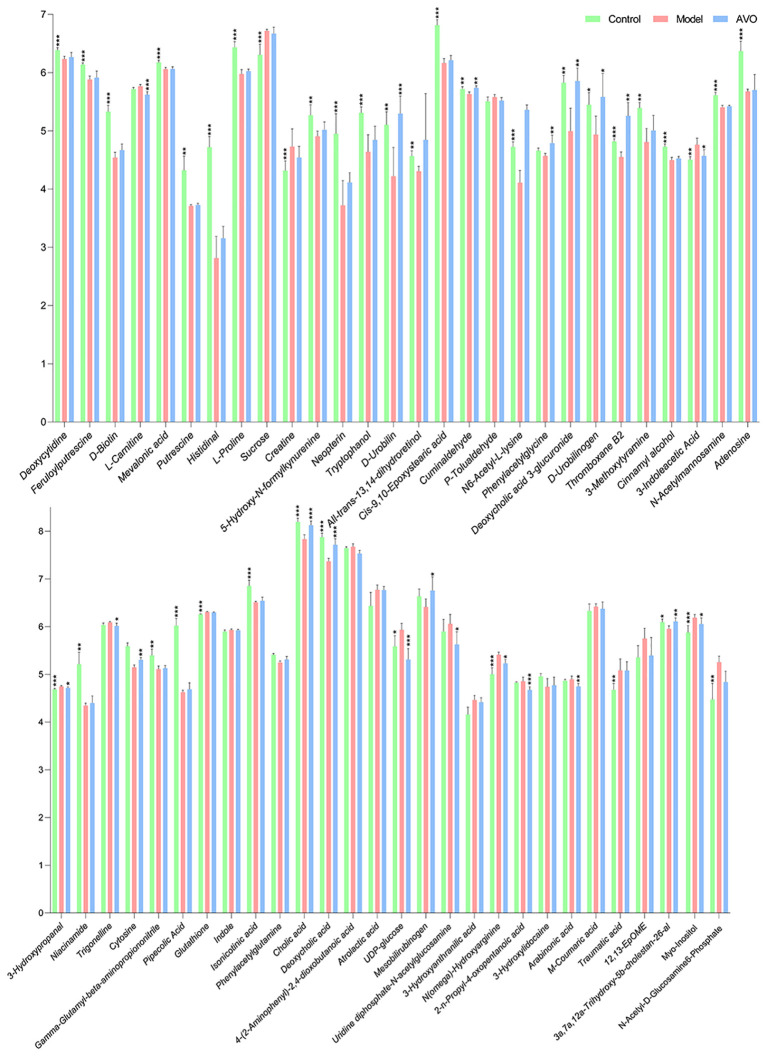
The abundance changes of 56 GM metabolites among the three groups (significance vs. the M group). *P ≤ 0.05, **P ≤ 0.01, ***P ≤ 0.001.

The KEGG enrichment analysis of all identified metabolites disclosed that these metabolites are involved in various KEGG pathways, including tryptophan metabolism, taurine and hypotaurine metabolism, sphingolipid signaling pathway, pyrimidine metabolism, and purine metabolism (**Figure 7A**). Subsequently, we analyzed all identified metabolites, and 56 GM metabolites meeting the requirements of statistical analysis were finally determined (**Table S2**). According to the KEGG database, 39 metabolites of 56 metabolites are involved in metabolic pathways; 14 metabolites of these metabolites are involved in the biosynthesis of secondary metabolites; 8 metabolites of these metabolites are involved in ABC transporters; 6 metabolites of these metabolites are involved in bile secretion; 6 metabolites of these metabolites involved in tryptophan metabolism. Furthermore, these metabolites are also involved in 97 other KEGG pathways. The enrichment analysis of 56 metabolites was performed and the result disclosed that ascorbate and aldarate metabolism, arginine and proline metabolism, tryptophan metabolism, galactose metabolism, amino sugar and nucleotide sugar metabolism were the 5 most affected metabolism pathways (**Figure 7B**). In addition, compared with the C group, the DSS challenge decreased 28 metabolites, including deoxycytidine, feruloylputrescine, *D*-Biotin, and so on, and increased 10 metabolites, including cortol, sucrose, creatine, and so on. Compared with the M group, AVO treatment decreased 9 metabolites, including *L*-carnitine, pelargonidin, 3-indoleacetic acid, and so on, and increased 10 metabolites, including *D*-urobilin, cuminaldehyde, phenylacetylglycine, and so on (**Figure 8**). These results suggested that AVO altered the GM metabolism of UC mice.

## 4 Discussion

UC incidence is rising worldwide and brings great economic pressure and pain to UC patients (1). To obtain a cure for UC, various chemically induced colitis models have been developed (17). Among these colitis models, the colitis model induced by DSS is widely used due to its simplicity and reproducibility. Just by adjusting the concentration and frequency of administration of DSS, we were able to construct various acute or chronic colitis models (18). In addition, the DSS-induced colitis model is widely used to investigate the role of GM and factors affecting GM on the occurrence and treatment of UC (17). Considering these advantages, we chose the colitis model induced by DSS as the UC experimental animal model in our study. In our study, compared with the C group, we observed significant weight loss, bloody diarrhea, and intestinal tissue damage in DSS-challenged mice and these symptoms are similar to human UC patients. In addition, the dysregulated immune responses are one of UC pathogenesis and colon tissue inflammation is also a common feature of UC patients and animals. Therefore, we evaluated the changes in immune-related cytokines. The previous study has shown that the disrupted IL-2 gene contributes to developing an inflammatory bowel disease similar to UC in humans and IL-2 and IL-4 were defined as anti-inflammatory cytokines which are negatively associated with the occurrence of UC (19, 20). In addition, INF-*γ* and IL-17, two proinflammatory cytokines, were positively associated with UC and the abnormally increased INF-*γ* and IL-17 have been observed in many UC animals and patients (21, 22). The result of our study was that the concentration of IL-2 and IL-4 was lower and the level of INF-*γ* and IL-17 was higher in the colon tissue of colitis mice. Based on these facts, we believed that we have successfully constructed the UC animal model and subsequent studies on AVO pharmacodynamics are of scientific significance. Compared with the M group, AVO treatment relieved bloody diarrhea and colon tissue damage and increased the concentration of IL-2 and IL-4. These results indicated that AVO has anti-colitis activity and provided a reasonable premise for our further exploration of the underlying mechanism by which AVO alleviates UC.

GM plays an important role in host homeostasis, especially intestinal homeostasis (12). To explore whether the bioactivity of AVO on UC is closely correlated to regulating GM, we detected the change in GM after the DSS challenge and AVO treatment. Multiple studies found that GM had some similar changes in UC animals and patients, including decreased microbial richness and diversity, the dysregulated GM overall structure, and the enriched or suppressed specific microorganisms (23). Similarly, in our study, the richness of GM was decreased and the GM overall structure was altered in colitis mice, as evidenced by the decreased Ace and Chao index, the separated hierarchical clustering, and the separated 3D PCoA plot. Although AVO didn’t alleviate DSS-induced reduction in GM richness, an increasing trend was observed. Additionally, AVO didn’t reverse the dysregulated GM overall structure. As shown in the community histogram, *Firmicutes* and *Bacteroidota* are the two largest microbial communities. In addition, DSS interference decreased *Verrucomicrobiota* and increased *Proteobacteria* compared with the C group mice. AVO treatment reversed the effect of DSS on *Verrucomicrobiota* and *Proteobacteria*. Although the change of *Verrucomicrobiota* and *Proteobacteria* reflected the intimate relationship between AVO and DSS, the proportion of *Verrucomicrobiota* and *Proteobacteria* accounted for only a small fraction of all detected microorganisms. More studies are needed to find out other possible microorganisms responsible for the anti-colitis activity of AVO. In summary, these results suggested that DSS may induce UC by changing the overall structure of GM, and the change of GM may be responsible for the anti-colitis activity of AVO.

We also dug deeper into the changes of GM at the genus level and expect to screen out specific microorganisms potentially responsible for the efficacy of AVO. The increased *Turicibacter* had been observed in many UC individuals, and *Turicibacter* was positively associated with inflammation (24, 25). In our study, *Turicibacter* was conspicuously increased after the DSS challenge, and oral AVO decreased *Turicibacter* which has been increased by DSS. In addition, the increased *Parasutterella* and *Erysipelatoclostridium* have been found in many UC animals and similar results were found in our experiments as well (8, 26). These results indicated that *Parasutterella* and *Erysipelatoclostridium* were positively related to the occurrence of UC. However, the increased *Parasutterella* and *Erysipelatoclostridium* induced by DSS stimulation were conspicuously decreased after AVO intervention. In addition, LEfSe showed that *Turicibacter*, *Parasutterella*, and *Erysipelatoclostridium* were the dominant species in the M group mice. These results suggested that AVO may restrain the growth of potentially harmful bacteria (*Turicibacter*, *Parasutterella*, and *Erysipelatoclostridium*) to improve UC.

*Enterorhabdus* was negatively associated with the concentration of proinflammatory cytokines, and the decreased *Enterorhabdus* has been observed in UC mice as well (27, 28). These facts indicated that *Enterorhabdus* play a positive role in intestinal homeostasis. In our study, the phenomenon that DSS-challenged mice displayed a depletion of *Enterorhabdus* in line with other studies (28, 29). However, AVO treatment significantly enriched *Enterorhabdus* compared with the M group. *Akkermansia* is a microorganism inhibiting the intestinal mucus layer (30). Due to these facts that *Akkermansia* can increase the number of mucins and reduce colonic inflammation (31), *Akkermansia* had been suggested to have a positive effect on maintaining intestinal barrier homeostasis. In our study, *Akkermansia* was significantly reduced after DSS intervention, and oral AVO can significantly enrich *Akkermansia* compared with the M group mice. *Parvibacter* may increase intestinal barrier function by enhancing the secretion of mucins and inhibiting inflammatory cytokines (27). In our study, *Parvibacter* was decreased in DSS-challenged UC mice, and AVO treatment significantly enriched *Parvibacter*. LEfSe showed that *Enterorhabdus* and *Parvibacter* were the dominant species in the C group mice and *Akkermansia* was the dominant species in the AVO group mice. These results suggested that AVO may enrich these potentially beneficial bacteria (*Enterorhabdus*, *Parvibacter*, and *Akkermansia*) to improve UC.

The importance of GM metabolites on intestine homeostasis had been emphasized by a large number of researchers (32, 33). Therefore, in addition to GM composition, the bioactivity of AVO on UC may be correlated to regulating GM metabolism. In our study, the PCA score plot suggested that DSS and AVO significantly affected the metabolites of the fecal sample, and 56 altered GM metabolites involved in 102 KEGG pathways were ultimately identified. The comparative analysis of differences between groups revealed that after the DSS challenge, 28 metabolites were decreased and 10 metabolites were increased. Additionally, AVO treatment decreased 9 metabolites and increased 10 metabolites compared with the M group. These results suggested that the improvement of AVO on UC may be closely correlated to regulating GM metabolism.

To find out the metabolism pathways that may be bound up with the anti-colitis effect of AVO, we provide an in-depth discussion of the relationship between modified metabolism pathways and UC. We observed significant alterations in amino acid metabolism in our study. These altered amino acid metabolism pathways include tryptophan metabolism, tyrosine metabolism, lysine degradation, glycine, arginine and proline metabolism. Among these metabolism pathways, the importance of tryptophan metabolism on intestine homeostasis has been emphasized by many researchers. Dietary-derived tryptophan can be metabolized into various tryptophan microbial derivatives by GM (34). These microbial derivatives can activate the AHR to maintain intestinal homeostasis (35, 36). In our study, compared with the C group, the abundance of 5-hydroxy-*N*-formylkynurenine and tryptophanol was decreased and that of 3-indoleacetic acid and 3-hydroxyanthranilic acid was increased. Compared with the M group, AVO decreased the level of 4-(2-aminophenyl)-2, 4-dioxobutanoic acid and 3-indoleacetic acid (**Figure 8**).

Bile acids (BAs) are a series of metabolites that are metabolized by GM after being secreted by the host into the intestine. Research disclosed that BAs participate in maintaining the integrity of intestine epithelial tight junctions, promote the formation of the mucus layer, and coordinate intestine immune homeostasis (33, 37). In addition, the dysregulated BAs profile has been discovered in many UC patients and animals, and the regulation of BAs metabolism had been recently proposed as the mechanism of bioactive components in treating UC (38–40). In our study, we observed that the abundance of cholic acid and deoxycholic acid was decreased in UC mice. However, AVO treatment effectively reversed the effect of DSS on the abundance of cholic acid and deoxycholic acid (**Figure 8**). In addition, primary BAs are secreted into the intestine in the form of bile salts along with bile (41). In our study, 7 of 56 GM metabolites are associated with the bile secretion KEGG pathway, including *L*-carnitine, deoxycholic acid 3-glucuronide, thromboxane B2, glutathione, cholic acid, deoxycholic acid (**Figure 8**).

In addition to tryptophan metabolism and BAs metabolism, more and more research recently disclosed that the microbial metabolites of retinol (vitamin A) play important role in intestinal immune homeostasis (42). Retinoic acid, one of the microbial metabolites of retinol, can maintain intestinal homeostasis through various mechanisms, such as maintaining the number of eosinophils which can suppress the production of INF-*γ* in the intestine, regulating protective CD8^+^ T cells, and regulating the production of immunoglobulin A (43–45). In the study, we observed that all-*trans*-13, 14-dihydroretinol, an intermediate in the process of retinol metabolism to retinoic acid (46), was decreased in the M group mice (**Figure 8**).

In our study, we observed the altered GM metabolism in UC mice and AVO oral administration mice (as evidenced by the PCA score plots). More importantly, the results of differential metabolite analysis and KEGG enrichment analysis showed that, among these altered metabolism pathways, many metabolism pathways play an important role in maintaining intestinal homeostasis. Although this study did not prove that the anti-colitis effect of AVO is the result of regulating GM metabolism, it still suggested that the anti-colitis effect of AVO is related to regulating GM metabolism. Our study also showed that several metabolic pathways may be related to the anti-colitis effect of AVO, including amino acid metabolism (especially tryptophan metabolism), BA metabolism, and retinol metabolism. However, based on our research, further work should be carried out to study whether AVO exerts its anti-colitis activity by regulating these metabolic pathways.

The ingredient characteristics of AVO showed that the main components of AVO are atractylone (78.82%). Atractylone is a sesquiterpene oxide-derivative in essential oils from AM, and also be found in essential oils from other plants, such as *Siparuna muricate*, *Nectandra salicina* (*lauraceae*), *Atractylodes lancea* (Thunb.) DC.*, Siparuna guianensiswas* (47–51). Atractylone has many attractive pharmacological effects, including anti-inflammatory (52, 53). A recent study disclosed that atractylone improve gastric ulcer by altering GM composition (increased *Mucispirillum*, *Parabacteroides*, *Bacteroides* and decreased *Bifidobacterium*) and various GM metabolites (tryptophan and BAs) of gastric ulcer rats (51). These results indicated that atractylone may be the major bioactivity component of AVO with the capacity of regulating GM composition and metabolism, but further studies should be undertaken to verify whether atractylone treatment can improve colitis by regulating GM.

## 5 Conclusions

**Figure 9:**
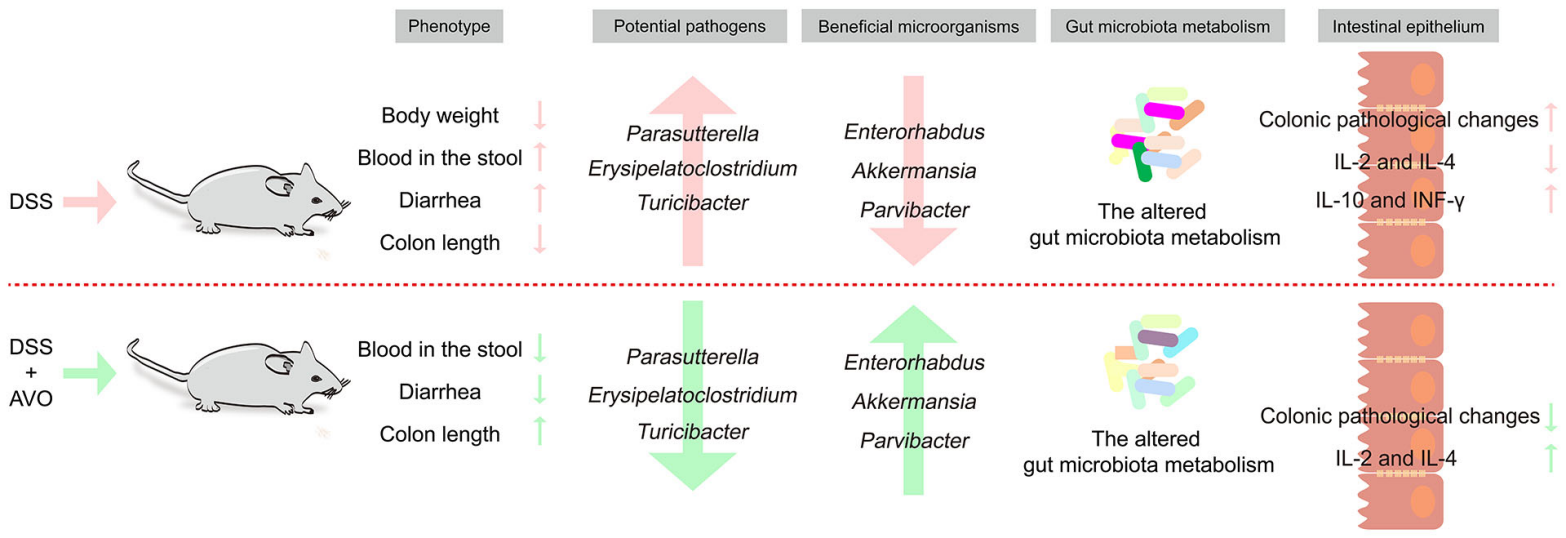
The summary diagram of the effects of AVO treatment on DSS-induced UC mice.

In our study, we first investigated whether AVO has a therapeutic effect on UC and the underlying mechanism. Our study disclosed that AVO can effectively alleviate bloody diarrhea, colon tissue damage, and colonic inflammation in UC mice. GM composition analysis of fecal samples disclosed that AVO restrained the growth of potentially harmful bacteria (*Turicibacter*, *Parasutterella*, and *Erysipelatoclostridium*) and enriched potentially beneficial bacteria (*Enterorhabdus*, *Parvibacter*, and *Akkermansia*). Fecal metabolomics showed that AVO can alter GM metabolism by regulating 56 metabolites involved in 102 KEGG pathways. Among these KEGG pathways, many metabolism pathways are closely related to intestinal homeostasis, such as amino acid metabolism (especially tryptophan metabolism), BAs metabolism, and retinol metabolism. In summary, our study suggested that AVO may be expected as novel prebiotics to treat UC *via* modulating GM and GM metabolism (**Figure 9**).

## Data availability statement

The datasets presented in this study can be found in online database NCBI Sequence Read Archive accession number PRJNA913668.

## Ethics statement

The animal study was reviewed and approved by Animal Ethics Committee in Chengdu University of Traditional Chinese Medicine.

## Author contributions

WF and CP conceived and proposed the idea. HC, JL, DZ, and JW performed the experiments. HC, JL, and DZ wrote and revised the manuscript. JL, YT, and CP checked the manuscript. All authors have read and approved the final manuscript.
