# Supplementary material for: Atractylodes macrocephala Koidz. volatile oil relieves acute ulcerative colitis via regulating gut microbiota and gut microbiota metabolism
Source: Front Immunol. 2023 May 2;14:1127785. doi: 10.3389/fimmu.2023.1127785 (PMC10187138; doi:10.3389/fimmu.2023.1127785)
Supplement: Supplementary file 1 [file DataSheet_1.docx]

***Atractylodes macrocephala* Koidz. volatile oil relieves acute ulcerative colitis via regulating gut microbiota and gut microbiota metabolism**

**Hao Cheng**^1^, **Dandan Zhang**^1^, **Jing Wu**^1^, **Juan Liu**^3^, **Yuzhu Tan**^1,2^, **Wuwen Feng**^1,2*^, **Cheng Peng**^1,2*^

^1^State Key Laboratory of Characteristic Chinese Medicine Resources in Southwestern China, School of Pharmacy, Chengdu University of Traditional Chinese Medicine, Chengdu, China

^2^The Ministry of Education Key Laboratory of Standardization of Chinese Herbal Medicine, School of Pharmacy, Chengdu University of Traditional Chinese Medicine, Chengdu, China

^3^Hospital of Chengdu University of Traditional Chinese Medicine, Chengdu 610072, China

^⁎^Corresponding author: (Wu-wen Feng, email: jiaoxiake-1@foxmail.com; Cheng Peng, email: pengchengcxy@126.com)


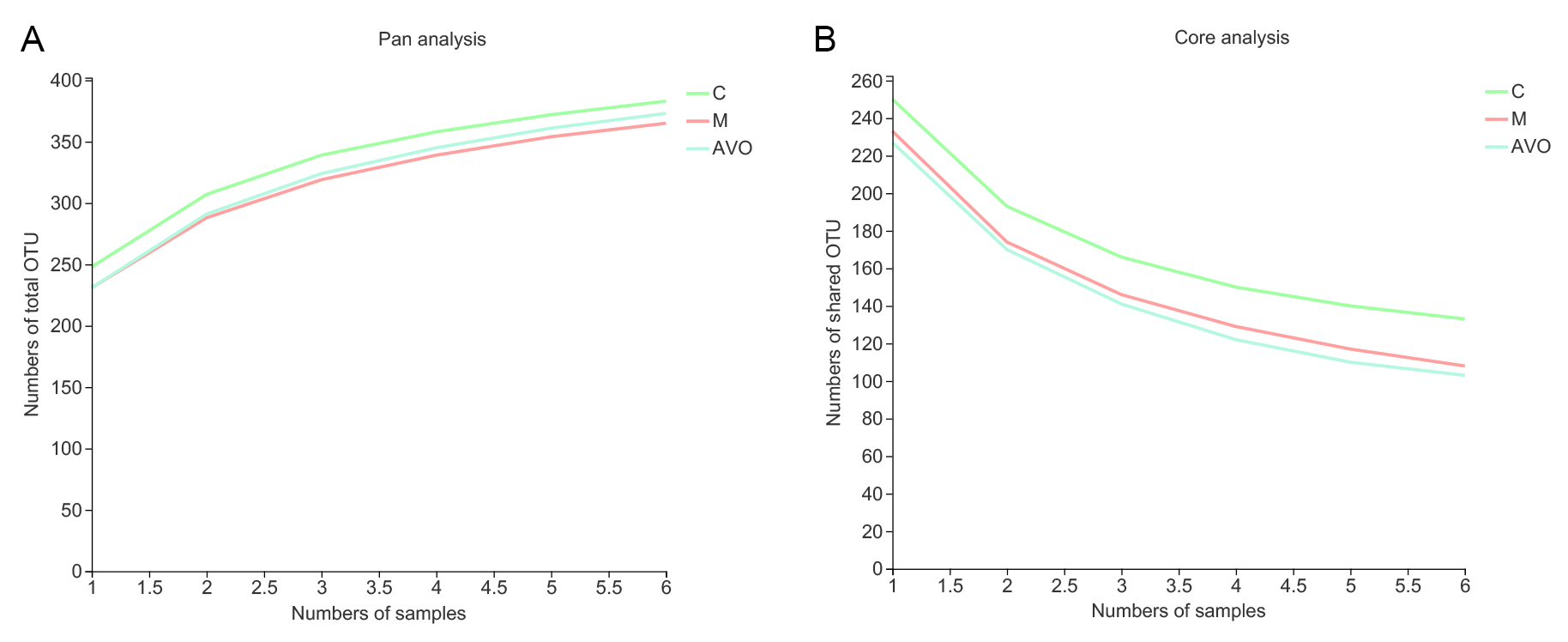


**FIGURE S1 |** The effect of AVO and DSS on gut microbiota overall structure in UC mice. **(A)** Pan analysis based on OTU level. **(B)** Core analysis based on the OUT level.


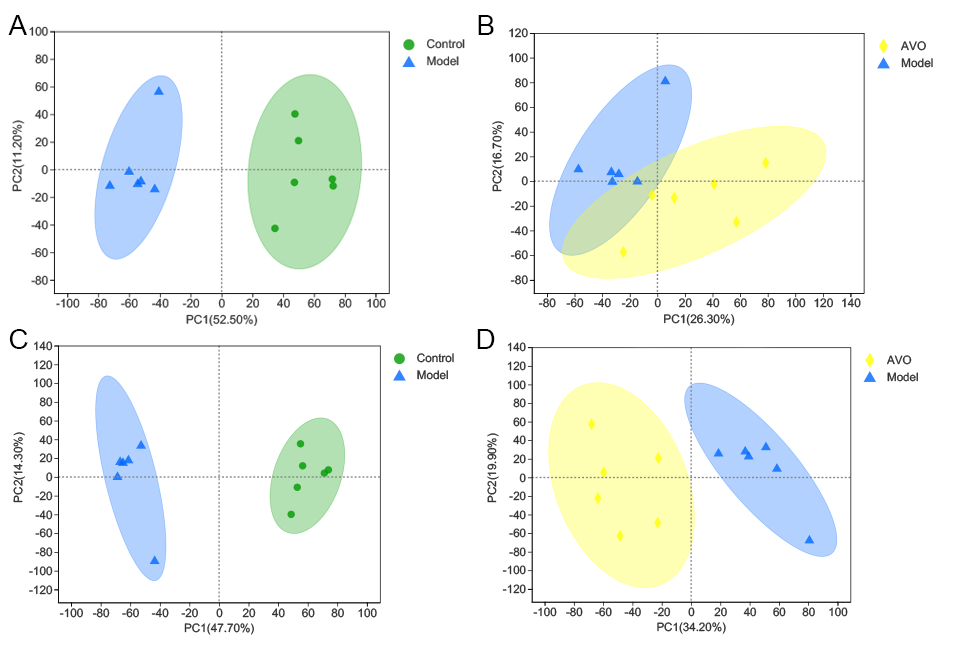


**FIGURE S2 |** DSS and AVO modulated gut microbiota metabolism. **(A)** PCA score plots shown the distribution between the C group and the M group in ESI positive ion mode. **(B)** PCA score plots shown the distribution between the M group and the AVO group in ESI positive ion mode. **(C)** PCA score plots shown the distribution of between the C group and the M group in ESI negative ion mode. **(D)** PCA score plots shown the distribution of between the M group and the AVO group in ESI negative ion mode.

**Table S1** **|** The mobile phase elution gradient method.

| Time (min) | Flow rate (ml/min) | A (%) | B (%) |
| --- | --- | --- | --- |
| 0 | 0.4 | 100 | 0 |
| 3.5 | 0.4 | 75.5 | 24.5 |
| 5 | 0.4 | 35 | 65 |
| 5.5 | 0.4 | 0 | 100 |
| 7.4 | 0.6 | 0 | 100 |
| 7.6 | 0.6 | 48.5 | 51.5 |
| 7.8 | 0.5 | 100 | 0 |
| 9 | 0.4 | 100 | 0 |
| 10 | 0.4 | 100 | 0 |

A: (95% water + 5% acetonitrile (with 0.1% formic acid); B: 47.5% acetonitrile + 47.5% isopropanol + 5% water (with 0.1% formic acid)

**Table S2 |** The abundance changes of 56 gut microbiota metabolites.

| Metabolites | KEGG ID | Control (mean ± SD) | M (mean ± SD) | AVO (mean ± SD) |
| --- | --- | --- | --- | --- |
| Deoxycytidine | C00881 | 6.3864 ± 0.0406 | 6.2354 ± 0.0452 | 6.2661 ± 0.0807 |
| Feruloylputrescine | C10497 | 6.1380 ± 0.0290 | 5.8810 ± 0.0605 | 5.9136 ± 0.1135 |
| *D*-Biotin | C00120 | 5.3289 ± 0.1096 | 4.5412 ± 0.0903 | 4.6677 ± 0.1063 |
| *L*-Carnitine | C00318 | 5.7173 ± 0.0283 | 5.7670 ± 0.0310 | 5.6199 ± 0.0539 |
| Mevalonic acid | C00418 | 6.1756 ± 0.0388 | 6.0602 ± 0.0312 | 6.0630 ± 0.0369 |
| Putrescine | C00134 | 4.3223 ± 0.2469 | 3.7119 ± 0.0217 | 3.7270 ± 0.0286 |
| Histidinal | C01929 | 4.7183 ± 0.1857 | 2.8147 ± 0.3720 | 3.1548 ± 0.2017 |
| *L*-Proline | C00148 | 6.4335 ± 0.0978 | 5.9764 ± 0.0754 | 6.0256 ± 0.0357 |
| Sucrose | C00089 | 6.3048 ± 0.1849 | 6.7159 ± 0.0311 | 6.6685 ± 0.1110 |
| Creatine | C00300 | 4.3158 ± 0.1685 | 4.7307 ± 0.3035 | 4.5403 ± 0.1940 |
| 5-Hydroxy-*N*-formylkynurenine | C05648 | 5.2684 ± 0.1810 | 4.9061 ± 0.0894 | 5.0166 ± 0.1370 |
| Neopterin | C05926 | 4.9531 ± 0.3412 | 3.7184 ± 0.4257 | 4.1137 ± 0.1679 |
| Tryptophanol | C00955 | 5.3095 ± 0.1038 | 4.6400 ± 0.2939 | 4.8429 ± 0.2378 |
| *D*-Urobilin | C05795 | 5.1047 ± 0.2241 | 4.2198 ± 0.4959 | 5.2961 ± 0.3035 |
| All-trans-13, 14-dihydroretinol | C15492 | 4.5665 ± 0.0906 | 4.3055 ± 0.0849 | 4.8453 ± 0.7945 |
| Cis-9, 10-epoxystearicacid | C19418 | 6.8142 ± 0.0961 | 6.1644 ± 0.0761 | 6.2126 ± 0.0814 |
| Cuminaldehyde | C06577 | 5.7164 ± 0.0412 | 5.6318 ± 0.0382 | 5.7376 ± 0.0370 |
| *P*-Tolualdehyde | C06758 | 5.5068 ± 0.0763 | 5.5797 ± 0.0468 | 5.5199 ± 0.0521 |
| N6-Acetyl-*L*-lysine | C02727 | 4.7253 ± 0.0860 | 4.1085 ± 0.2121 | 5.3603 ± 0.0867 |
| Phenylacetylglycine | C05598 | 4.6596 ± 0.0447 | 4.5711 ± 0.0427 | 4.7890 ± 0.1380 |
| Deoxycholic acid-3-glucuronide | C03033 | 5.8258 ± 0.1265 | 4.9951 ± 0.3946 | 5.8560 ± 0.2224 |
| *D*-Urobilinogen | C05791 | 5.4490 ± 0.2108 | 4.9373 ± 0.3180 | 5.5852 ± 0.4007 |
| Thromboxane B2 | C05963 | 4.8188 ± 0.0472 | 4.5508 ± 0.0872 | 5.2578 ± 0.2286 |
| 3-Methoxytyramine | C05587 | 5.3945 ± 0.0903 | 4.8079 ± 0.2324 | 5.0058 ± 0.2601 |
| Cinnamylalcohol | C02394 | 4.7287 ± 0.0467 | 4.4986 ± 0.0461 | 4.5251 ± 0.0376 |
| 3-Indoleacetic acid | C00954 | 4.5054 ± 0.0526 | 4.7620 ± 0.1128 | 4.5700 ± 0.1063 |
| *N*-Acetylmannosamine | C00645 | 5.6111 ± 0.0508 | 5.4036 ± 0.0355 | 5.4229 ± 0.0176 |
| Adenosine | C00212 | 6.3691 ± 0.1704 | 5.6769 ± 0.0402 | 5.7016 ± 0.2670 |
| 3-Hydroxypropanal | C00969 | 4.6897 ± 0.0178 | 4.7504 ± 0.0166 | 4.7208 ± 0.0205 |
| Niacinamide | C00153 | 5.2118 ± 0.2512 | 4.3468 ± 0.0505 | 4.3997 ± 0.1473 |
| Trigonelline | C01004 | 6.0369 ± 0.0417 | 6.0902 ± 0.0209 | 6.0152 ± 0.0591 |
| Cytosine | C00380 | 5.5912 ± 0.0717 | 5.1492 ± 0.0502 | 5.3057 ± 0.0477 |
| Gamma-Glutamyl-beta-aminopropiononitrile | C06114 | 5.3992 ± 0.1314 | 5.1126 ± 0.0657 | 5.1312 ± 0.0573 |
| Pipecolic acid | C00408 | 6.0218 ± 0.1500 | 4.6272 ± 0.0414 | 4.6906 ± 0.1332 |
| Glutathione | C00051 | 6.2607 ± 0.0105 | 6.3075 ± 0.0142 | 6.3007 ± 0.0097 |
| Indole | C00463 | 5.8998 ± 0.0346 | 5.9315 ± 0.0237 | 5.9239 ± 0.0200 |
| Isonicotinic acid | C07446 | 6.8533 ± 0.1210 | 6.5098 ± 0.0231 | 6.5440 ± 0.0747 |
| Phenylacetylglutamine | C04148 | 5.4110 ± 0.0280 | 5.2495 ± 0.0335 | 5.3132 ± 0.0626 |
| Cholic acid | C00695 | 8.1950 ± 0.0757 | 7.8347 ± 0.0895 | 8.1280 ± 0.0866 |
| Deoxycholic acid | C04483 | 7.8748 ± 0.0866 | 7.3684 ± 0.0646 | 7.7183 ± 0.1236 |
| 4-(2-Aminophenyl)-2, 4-dioxobutanoic acid | C01252 | 7.6456 ± 0.0293 | 7.6746 ± 0.0637 | 7.5317 ± 0.0664 |
| Atrolactic acid | C05584 | 6.4341 ± 0.2839 | 6.7735 ± 0.1011 | 6.7679 ± 0.0755 |
| UDP-glucose | C00029 | 5.5885 ± 0.2248 | 5.9339 ± 0.1362 | 5.3130 ± 0.2301 |
| Mesobilirubinogen | C05790 | 6.6351 ± 0.1534 | 6.4142 ± 0.1671 | 6.7584 ± 0.2861 |
| Uridinediphosphate-*N*-acetylglucosamine | C00043 | 5.8996 ± 0.2547 | 6.0629 ± 0.1955 | 5.6283 ± 0.2653 |
| 3-Hydroxyanthranilic acid | C00632 | 4.1590 ± 0.1550 | 4.4655 ± 0.0950 | 4.4188 ± 0.0879 |
| *N*-Hydroxyarginine | C05933 | 5.0034 ± 0.1380 | 5.4109 ± 0.0575 | 5.2319 ± 0.0811 |
| 2-n-Propyl-4-oxopentanoic acid | C16655 | 4.8254 ± 0.0217 | 4.8572 ± 0.0864 | 4.6742 ± 0.0681 |
| 3-Hydroxylidocaine | C16560 | 4.9608 ± 0.0553 | 4.7394 ± 0.1754 | 4.7727 ± 0.1727 |
| Arabinonic acid | C00878 | 4.8695 ± 0.0266 | 4.8999 ± 0.0679 | 4.7504 ± 0.0590 |
| M-Coumaric acid | C12621 | 6.3322 ± 0.1441 | 6.4226 ± 0.0580 | 6.3777 ± 0.1398 |
| Traumaticacid | C16308 | 4.6737 ± 0.1341 | 5.0850 ± 0.2399 | 5.0816 ± 0.1844 |
| 12, 13-EpOME | C14826 | 5.3564 ± 0.2484 | 5.7518 ± 0.2160 | 5.3954 ± 0.3766 |
| 3a, 7a, 12a-Trihydroxy-5b-cholestan-26-al | C01301 | 6.0953 ± 0.0662 | 5.9547 ± 0.0624 | 6.1087 ± 0.0768 |
| Myo-Inositol | C00137 | 5.8788 ± 0.1466 | 6.1909 ± 0.0652 | 6.0554 ± 0.1287 |
| *N*-Acetyl-*D*-Glucosamine6-Phosphate | C00357 | 4.4715 ± 0.3428 | 5.2581 ± 0.1261 | 4.8422 ± 0.2274 |
